# Compression-Complexity Measures for Analysis and Classification of Coronaviruses

**DOI:** 10.3390/e25010081

**Published:** 2022-12-31

**Authors:** Naga Venkata Trinath Sai Munagala, Prem Kumar Amanchi, Karthi Balasubramanian, Athira Panicker, Nithin Nagaraj

**Affiliations:** 1 Department of Electronics and Communication Engineering, Amrita School of Engineering, Coimbatore, Amrita Vishwa Vidyapeetham, Ettimadai 641112, Tamil Nadu, India; 2 Consciousness Studies Programme, National Institute of Advanced Studies, Bengaluru 560012, Karnataka, India

**Keywords:** compression-complexity measures, Effort-to-Compress complexity, Lempel-Ziv complexity, distance measure, machine learning, COVID-19

## Abstract

Finding a vaccine or specific antiviral treatment for a global pandemic of virus diseases (such as the ongoing COVID-19) requires rapid analysis, annotation and evaluation of metagenomic libraries to enable a quick and efficient screening of nucleotide sequences. Traditional sequence alignment methods are not suitable and there is a need for fast alignment-free techniques for sequence analysis. Information theory and data compression algorithms provide a rich set of mathematical and computational tools to capture essential patterns in biological sequences. In this study, we investigate the use of compression-complexity (Effort-to-Compress or ETC and Lempel-Ziv or LZ complexity) based distance measures for analyzing genomic sequences. The proposed distance measure is used to successfully reproduce the phylogenetic trees for a mammalian dataset consisting of eight species clusters, a set of coronaviruses belonging to group I, group II, group III, and SARS-CoV-1 coronaviruses, and a set of coronaviruses causing COVID-19 (SARS-CoV-2), and those not causing COVID-19. Having demonstrated the usefulness of these compression complexity measures, we employ them for the automatic classification of COVID-19-causing genome sequences using machine learning techniques. Two flavors of SVM (linear and quadratic) along with linear discriminant and fine K Nearest Neighbors classifer are used for classification. Using a data set comprising 1001 coronavirus sequences (causing COVID-19 and those not causing COVID-19), a classification accuracy of 98% is achieved with a sensitivity of 95% and a specificity of 99.8%. This work could be extended further to enable medical practitioners to automatically identify and characterize coronavirus strains and their rapidly growing mutants in a fast and efficient fashion.

## 1. Introduction

Pandemics such as the ongoing COVID-19 (caused by SARS-CoV-2) pandemic that leads to enormous loss of life globally can only be controlled by vaccines or a very effective antiviral treatment. Finding a vaccine or specific antiviral treatment for such a global pandemic of virus diseases requires rapid analysis, annotation, and evaluation of metagenomic libraries to enable quick and efficient screening of nucleotide sequences. Traditional sequence alignment methods are not suitable since they are computationally intensive and cannot be easily scaled up as the number of sequences increases. Thus, there is a need for fast alignment-free techniques for sequence analysis [1,2]. Information theory and data compression algorithms provide a rich set of mathematical and algorithmic/computational tools to capture essential patterns in data that could be used for matching nucleotide sequences.

Genome sequences are inherently described by character strings and are hence amenable to mathematical and computational techniques for extracting information. Exactly what information is being sought from such character strings depends on the string itself and the domain as well as the kind of application. Some targets of interest for analyzing genome sequences include:

- Identifying various genes that constitute the genome.
- Identifying the origin of the genome sequence.
- Understanding the information content present in the coding and non-coding regions.
- Reconstructing the phylogenetic tree to study evolutionary patterns.
- Automatic classification and identification of unknown genome sequences.

An important objective is to automate the above tasks so that a large number of sequences can be quickly, robustly, and efficiently analyzed (as one of the steps in the endeavor for finding a vaccine).

A cursory glance at these character strings does not tell us much about how they can be used for these applications. However, a harmonious blending of complexity analysis with the field of information theory provides deep insight in this regard. Application of complexity measures on these information-bearing character strings may reveal many surprising features that generally cannot be discerned by intuition or visual inspection of the data alone.

In this study, we propose compression-complexity based distance measures for the analysis of genomic sequences. To validate the efficacy of this distance measure, we first apply it to the mitochondrial DNA sequences of primates belonging to eight different species clusters and recreate a phylogenetic tree showing these clusters accurately. Subsequently, the distance measure is applied to a group of Severe Acute Respiratory Syndrome (SARS-CoV-1) coronaviruses and also to a group of SARS-CoV-2 viruses to successfully reconstruct their phylogenetic trees, grouping the viruses correctly.

Having demonstrated the usefulness of these compression-complexity measures, we employ them for the automatic classification of SARS-CoV-2 viruses using machine learning techniques. The compression-complexity measures extracted from the sequences are passed as features to machine learning algorithms (linear and quadratic SVM, linear discriminant, and fine KNN) for classification.

The paper is organized as follows. In Section 2, a brief overview of genetic sequences and methods of analysis are described. Section 3 deals with the materials used (genome primary sequence data with their details) and the methods proposed in this study. Machine learning methods for classification of SARS-CoV-2 sequences are also introduced. This is followed by results and discussion in Section 4 and the paper concludes in Section 5 with suggestions for future research.

## 2. Genomic Sequences and Comparison

### 2.1. Genome and Gene

The total DNA content (RNA for viruses) of an organism is known as the genome, thus representing the entire information coded in a cell, while a gene represents a section of the DNA that codes for RNA or protein. A genome consists of a sequence of multiple genes interspersed with non-coding sequences of nucleic bases [3].

### 2.2. Genome Sequence Comparison

Genome data classification comes under the broad field of bioinformatics, an established multidisciplinary field for over three decades, encompassing physical and life sciences, computer science, and engineering. Many fundamental problems in the fields of medicine and biology are being tackled using the tools of bioinformatics. The main requirement for accomplishing such tasks is the availability of sequenced genome data. This has been the focus of researchers for the past few decades and efforts have been made by the National Institutes of Health (NIH) to establish Genbank^®^ (http://www.ncbi.nlm.nih.gov/genbank (accessed on 4 October 2022)), a genetic sequence database containing an annotated collection of all publicly available DNA sequences. Ever since its inception in 1982, there has been an exponential rise in the number of sequences in Genbank. This has provided the required resources for researchers and industry people alike for delving into the field of bioinformatics.

Among the various aspects involved in bioinformatics, one key element is sequence comparison or analysis of sequence similarity [4]. This is used in database searching, sequence identification and classification, phylogenetic tree (also called an evolutionary tree, is a tree diagram that shows the evolutionary relationships among different species according to the composition of their genes) creation, gene annotation and evolutionary modeling. Since it is impossible to recreate or simulate past evolutionary events, computational and statistical methods for comparison of nucleotide and protein sequences are used for these kinds of studies [1,5].

There are two kinds of sequence comparison methods:

- *Alignment-based methods*: These involve either shifting or insertion of gaps in sequences for the optimal alignment of two or more sequences [6,7]. The alignment involves selected scoring systems and gives high accuracy, but they are computationally intensive and consume huge memory.
- *Alignment-free methods*: These are computationally less intensive methods that consider the genome sequences as character strings and use distance-based methods involving frequency and distribution of bases [8,9,10,11,12]. Our focus in this paper is on alignment-free methodology, especially on using compression-complexity measures for sequence comparisons.

Sequence comparison and genome data classification got a boost in the early 1990s with the use of data compression algorithms that can identify regularities in sequences [13]. They provided a means to define distances between two sequences that greatly aided in the comparison of sequences. The history behind the usage of data compression algorithms in this field has been elucidated by Otu and Sayood in [13]. We succinctly summarize that history here.

The first attempt at using data compression for phylogenetic tree construction was by Grumbach et al. in [14]. They explored the idea of compressing a sequence *S* using a sequence *Q*, where the degree of compression obtained by doing so would be an indicator of the distance between them. Although their definition was not mathematically valid, it set a platform for researchers to explore this area. Varre et al. [15] defined a transformation distance when sequence *Q* is transformed to sequence *S* by various mutations like segment-copy, segment-reverse copy, and segment-insertion. Li et al. [16] define a relative distance measure by using a compression algorithm called GenCompress [17] that is based on approximate repeats in DNA sequences. Using the concept of Kolmogorov complexity, the compression algorithm has been used to propose a distance between sequences *S* and *Q*. However, Kolmogorov complexity, [18] being an algorithmic measure of information and a theoretical limit, cannot be directly computed but only approximately estimated [19]. Hence it is not an optimum choice as a complexity measure. Even though the idea of relative distance is an efficient one, GenCompress is a complicated algorithm that is computationally intensive. To overcome the above-mentioned difficulties, Otu and Sayood [13] proposed similar but computationally simpler relative distance measures based on the Lempel-Ziv (LZ) [20] complexity measure. Given two sequences *S* and *Q*, sequences SQ and QS are formed by concatenation (*Q* is appended at the end of the sequence *S* to yield the new sequence SQ). These four sequences are used to define four distance measures using the LZ complexity measure, as given below:

d(S,Q)=max{LZ(SQ)−LZ(S),LZ(QS)−LZ(Q)}.

To account for the effect of the length of the sequence, a normalized measure is defined as follows:

$$d\left(S,Q\right)=\frac{max\{LZ(SQ)-LZ(S),LZ(QS)-LZ(Q)\}}{max\{LZ(S),LZ(Q)\}}.$$

A third distance metric based on *sum distance* is defined as follows:

d(S,Q)=LZ(SQ)−LZ(S)+LZ(QS)−LZ(Q).

Finally, the normalized version of the sum distance is defined as:

$$d\left(S,Q\right)=\frac{LZ(SQ)-LZ(S)+LZ(QS)-LZ(Q)}{LZ(SQ)}.$$

Using these distance measures on mtDNA (mitochondrial DNA) samples of a wide range of eutherans (placental mammals), they have successfully re-created phylogenetic trees showing the evolutionary patterns. Other researchers have used these and slight variants of these measures to identify families of coronaviruses, mammals, vertebrates, and salmons. Interested readers are referred to [21,22,23,24,25,26,27,28] for further details on these. Apart from these complexity-based measures, distance measures using Markov chain models [29,30,31] and measures of probability [32,33,34] have also been proposed for the study of genome identification.

## 3. Materials and Methods

In this section, we provide details of the datasets as well as the methods used in this study.

### 3.1. Genome Sequences Used in This Study

Genome data from mammals and various types of coronaviruses, obtained from Genbank database are used for the analysis. These are described below.

#### 3.1.1. Mammalian Sequences

The first dataset we considered was mitochondrial genomes (mtDNA) of 41 mammals grouped into 8 species clusters as shown in Table S1 (Supplementary Resource 1) [35].

#### 3.1.2. Coronaviruses (SARS-CoV-1)

For our analysis, we use genome sequences of the following viruses:

- 15 SARS-CoV-1 coronaviruses
- 15 Non-SARS-CoV-1 coronaviruses belonging to Groups I, II and III coronaviruses

For complete details of the above sequences with accession numbers, please refer to Table S2 (Supplementary Resource 1).

#### 3.1.3. SARS-CoV-2 (COVID-19 Causing Corona Viruses)

For the third set of analyses, we consider 30 COVID-1 causing coronavirus sequences and 30 coronavirus sequences not causing COVID-19 (consisting of alpha, beta, gamma, and deltacoronaviruses). For complete details of the above sequences with accession numbers, please refer to Table S3 (Supplementary Resource 1).

### 3.2. Mathematical and Computational Methods Used in This Study

#### 3.2.1. Compression Complexity Measures: Lempel–Ziv (LZ) and Effort-to-Compress (ETC)

For measuring the complexity of the nucleotide sequences, we have used Lempel-Ziv (LZ [20]) and Effort-to-Compress (ETC [36]) complexity measures. Lempel–Ziv complexity (LZ), a popular and widely used complexity measure, estimates the degree of compressibility of an input sequence. Effort-to-Compress, a relatively recent complexity measure, determines the number of steps required by the Non-Sequential Recursive Pair Substitution Algorithm to compress the input sequence to a constant sequence (or a sequence of zero entropy) [37]. It should be noted that both LZ and ETC are complexity measures derived from lossless data compression algorithms (hence we term them compression-complexity measures). Further, ETC consistently performs better than LZ in several applications as shown in recently published literature [38,39,40]. For details on how to compute LZ and ETC on actual input sequences, we refer the readers to Section S1 of supplementary information (Supplementary Resource 2).

#### 3.2.2. Distance Measure

We use a distance measure which is computed using a compression-complexity measure (LZ or ETC). Let us say that we have genome sequences of two viruses $V_{1}$ and $V_{2}$. Firstly, we form new sequences $V_{1}V_{2}$ and $V_{2}V_{1}$ by concatenation (AB is the new sequence obtained by simply concatenating sequence *B* at the end of sequence *A*). We then compute the complexity measures $ETC(V_{1})$, $ETC(V_{2})$, $ETC(V_{1}V_{2})$ and $ETC(V_{2}V_{1})$ (similarly for LZ). In line with what has been used by Otu and Sayood [13], the distance measure is given by the average of the relative distances between the complexity values of the two concatenated sequences $V_{1}V_{2}$ and $V_{2}V_{1}$. Mathematically, they are described as:

$$d_{LZ}\left(V_{1},V_{2}\right)=\frac{\left(LZ\left(V_{1}V_{2}\right)\;-\;LZ\left(V_{1}\right)\right)\;+\;\left(LZ\left(V_{2}V_{1}\right)\;-\;LZ\left(V_{2}\right)\right)}{2},$$

(1) $$d_{ETC}\left(V_{1},V_{2}\right)=\frac{\left(ETC\left(V_{1}V_{2}\right)\;-\;ETC\left(V_{1}\right)\right)\;+\;\left(ETC\left(V_{2}V_{1}\right)\;-\;ETC\left(V_{2}\right)\right)}{2}.$$

Note that the above distances will always be non-negative and symmetric (d(A,B)≥0, d(A,A)=0 and d(A,B)=d(B,A). The triangle inequality is also likely to hold).

#### 3.2.3. Machine Learning Algorithms Used in the Study

Various AI techniques are being currently used for COVID-19 related data analytics [41,42,43,44,45,46]. In this work, machine learning (ML) is used for classifying SARS-CoV-2 viruses from other coronaviruses. Since training of ML algorithms is data-intensive, we have used a total of 1001 sequences of coronaviruses (all sequences were obtained from GISAID (https://www.gisaid.org) (accessed on 4 October 2022)) belonging to alpha (131 sequences), beta (130 sequences), gamma (130 sequences) and delta (147 sequences) coronaviruses and SARS-CoV-2 (436 sequences).

The ML algorithms used for classification were support vector machines (SVM), linear (LSVM) and quadratic kernels (QSVM), fine K-nearest neighbors classifier (FKNN) using Euclidean distance with five nearest neighbors and linear discriminant algorithm (LD). A brief description of these methods can be found in Section S2 of supplementary information (Supplementary Resource 2). The Statistics and Machine Learning Toolbox^TM^ from MATLAB^®^ ver. R2021a was used for the analysis. To evaluate the system, analysis was done with a 10-fold cross-validation scheme, using a 90–10% dataset splitting (90% of the dataset for training and 10% for testing).

## 4. Results and Discussion

**Figure 1:**
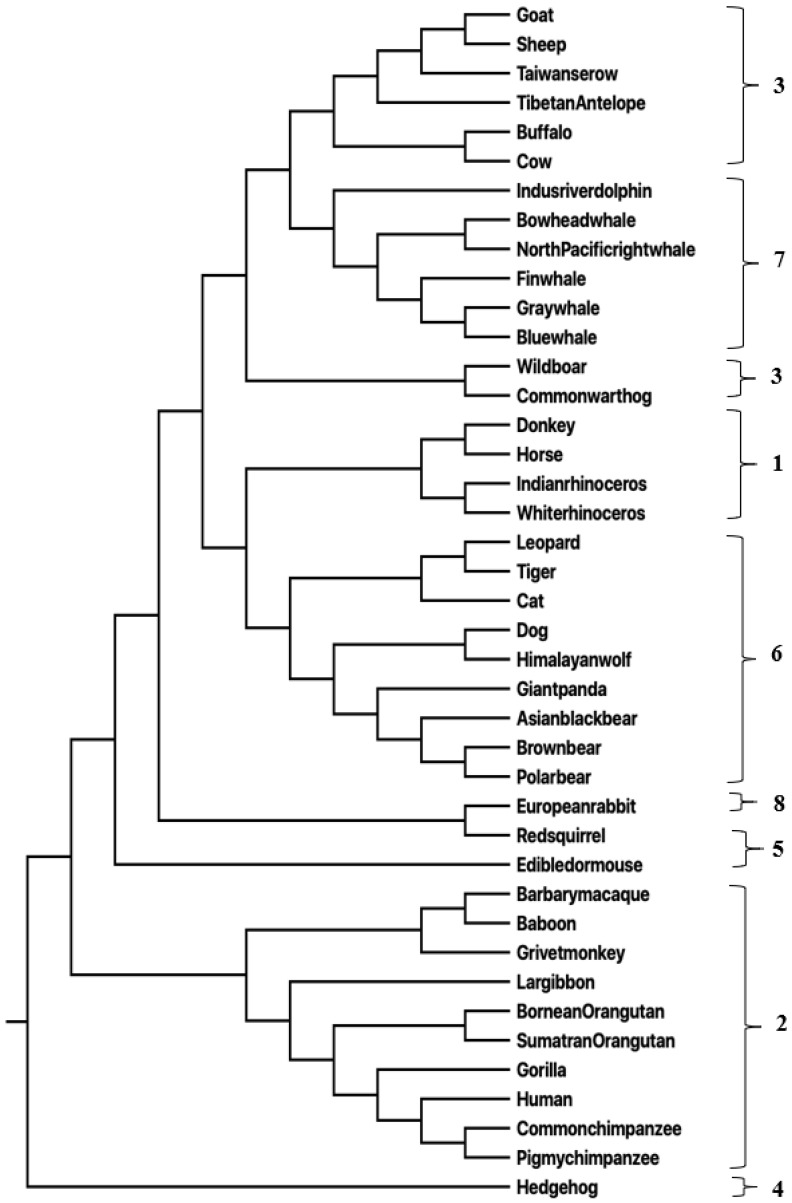
Phylogenetic tree for mammals generated using ETC-based distance measure.

For the mammalian mitochondrial genomes, the phylogenetic tree that was obtained with the distance measure using the ETC measure (Equation (1)) is depicted in Figure 1.

The pairwise distance values were fed to MEGA [47] to obtain the phylogenetic tree. The phylogenetic tree corresponding to LZC can be found in Figure S1 (Supplementary Resource 1). Both these phylogenetic trees closely match the tree (in terms of groupings) obtained in [35].

**Figure 2:**
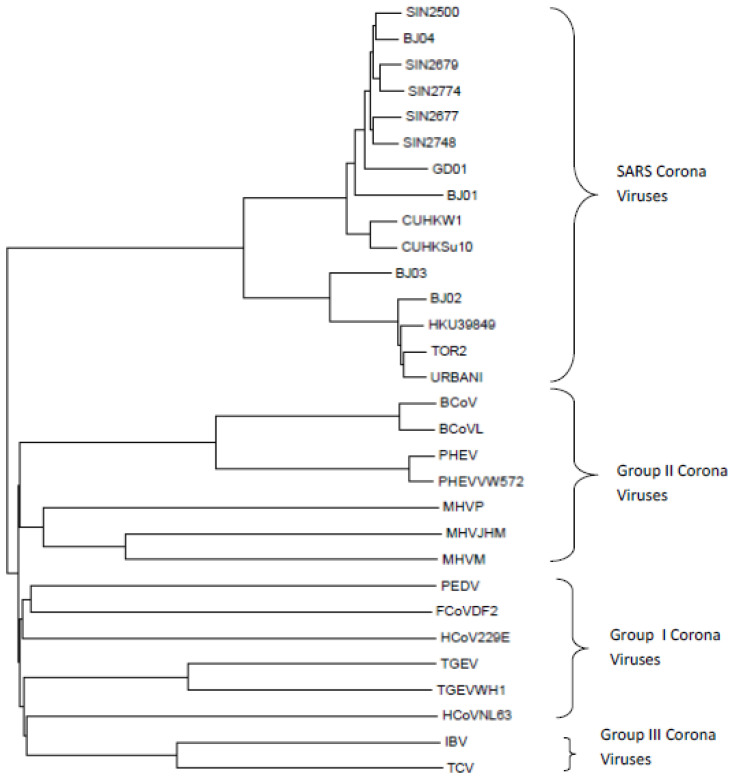
Phylogenetic tree generated for coronaviruses (SARS-CoV-1 and non-SARS-CoV-1) with ETC based distance measure.

**Figure 3:**
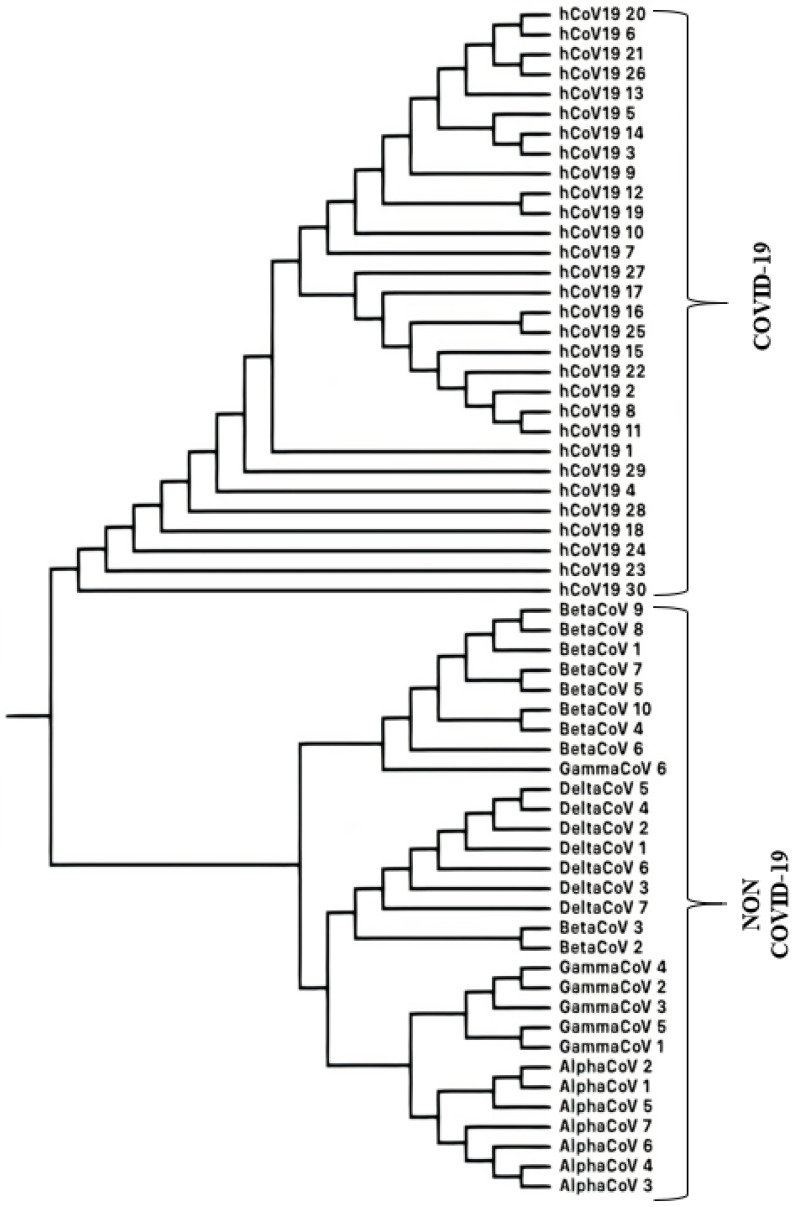
Phylogenetic tree generated for coronaviruses causing COVID-19 (SARS-CoV-2) and those not causing COVID-19 (non-SARS-CoV-2) using ETC based distance measure.

Having validated the distance measure on the mammalian dataset, we applied it to SARS-CoV-1 and non-SARS-CoV-1 coronaviruses and SARS-CoV-2 and non-SARS-CoV-2 coronaviruses. The phylogenetic trees thus obtained (using ETC) are shown in Figure 2 and Figure 3.

It can be seen in Figure 2 that each of the Group I, Group II, Group III coronaviruses, and SARS-CoV-1 coronaviruses form distinct groups separate from each other. The same can be seen in the LZC phylogenetic tree as shown in Figure S2 (Supplementary Resource 1). Similarly in Figure 3, the COVID-19 and non-COVID-19 causing coronaviruses form separate distinct groups. Except for GammaCoV 6 coronavirus, within the sequences not causing COVID-19, we find that alphacoronaviruses, betacoronaviruses, gammacoronaviruses and deltacoronaviruses are all clustered in distinct groups. In the corresponding phylogenetic tree generated by the LZC distance measure as shown in Figure S3 (Supplementary Resource 1), COVID-19 and non-COVID-19-causing coronaviruses also fall into separate groups. However, among the sequences not causing COVID-19, there are three exceptions. Different versions of the phylogenetic tree for the COVID-19 and non-COVID-19-causing coronaviruses are shown in Figures S4 and S5 (Supplementary Resource 1).

### Classification of SARS-CoV-2 Sequences

In this section, we analyze the performance of various machine learning algorithms for the classification of SARS-CoV-2 (COVID-19) sequences. Table 1, Table 2 and Table 3 summarize the results obtained for the classification of SARS-CoV-2 (COVID-19) vs. non-SARS-CoV-2 coronaviruses using LSVM, QSVM, LD and FKNN algorithms with ETC and LZC values as features.

Fine KNN outperforms LSVM, QSVM and LD algorithms in terms of F1-score and accuracy. It can be seen that, using ETC or LZC alone as a feature gives detection accuracies in the range of 80–90% while, using a combination of both gives an accuracy of more than 90%. This shows that combining features from multiple compression-complexity measures can significantly boost the classification accuracies of SARS-CoV-2 sequences.

## 5. Conclusions

Compression-complexity measures such as LZ and ETC which are based on lossless compression algorithms are good candidates for developing fast alignment-free methods for genome sequence analysis, comparison, and identification. The main reason for this is their ability to characterize and analyze information in biological sequences with contiguous segments. The distance measure based on LZ and ETC showed excellent recreation of phylogenetic trees for mammalian mtDNA sequences, SARS-CoV-1 coronaviruses, and SARS-CoV-2 viruses. Compression-complexity measures also serve as excellent features for the automatic classification of COVID-19-causing coronaviruses using machine learning algorithms. Our results demonstrate that combining ETC and LZC yields very high classification accuracies: 98% for the fine K-Nearest Neighbors classifier. Thus, compression-complexity measures such as ETC and LZC provide an efficient alternative to standard methods for genome analysis and classification of coronaviruses.

## Figures and Tables

**Table 1:** Machine learning for classification of SARS-CoV-2 sequences using LZC as feature. Accuracy is in percentage.

ML Methods	Accuracy	Precision	Sensitivity	Specificity	F1-Score
**LSVM**	89	0.98	0.82	0.98	0.89
**QSVM**	90	1	0.82	1	0.90
**LD**	86	1	0.80	1	0.88
**FKNN**	92	0.96	0.89	0.96	0.92

**Table 2:** Machine learning for classification of SARS-CoV-2 sequences using ETC as feature. Accuracy is in percentage.

ML Methods	Accuracy	Precision	Sensitivity	Specificity	F1-Score
**LSVM**	80	0.74	0.90	0.70	0.81
**QSVM**	83	0.84	0.74	0.89	0.79
**LD**	84	0.81	0.85	0.83	0.83
**FKNN**	88	0.95	0.79	0.96	0.86

**Table 3:** Machine learning for classification of SARS-CoV-2 sequences using both LZC and ETC as features. Accuracy is in percentage.

ML Methods	Accuracy	Precision	Sensitivity	Specificity	F1-Score
**LSVM**	92	0.98	0.89	0.97	0.93
**QSVM**	95	1	0.90	1	0.95
**LD**	87	1	0.79	1	0.88
**FKNN**	98	1	0.96	1	0.98

## Data Availability

The data used in the analysis is publicly available and can be downloaded from https://www.ncbi.nlm.nih.gov/ (accessed on 4 October 2022).

## Supplementary Materials

The following are available online at https://www.mdpi.com/article/10.3390/e25010081/s1.

## Abbreviations

The following abbreviations are used in this manuscript:

ETC	Effort-to-Compress complexity
LZC	Lempel ziv complexity
LSVM	Linear Support Vector Machine
QSVM	Quadratic Support Vector Machine
LD	Linear Discriminant
FKNN	Fine K-Nearest Neighbors
