# Supplementary material for: Compression-Complexity Measures for Analysis and Classification of Coronaviruses
Source: Entropy (Basel). 2022 Dec 31;25(1):81. doi: 10.3390/e25010081 (PMC9857615; doi:10.3390/e25010081)
Supplement: Supplementary file 1 [file entropy-25-00081-s001.zip › Supplementary_Resource 1.pdf]

Supplementary Information-1 for 'Compression-complexity measures for analysis and classification of coronaviruses'

December 29, 2022

Dataset details and Phylogenetic trees

The datasets utilised in this work, and sample phylogenetic trees generated, are presented below.

Table S1: Mitochondrial genomes (mtDNA) of 41 mammals grouped into 8 species clusters

Order	Scientific name	Common name	Accession Number
Perissodactyla	Rhinoceros unicornis	Indian Rhinoceros	X97336.1
	Ceratotherium simum	White Rhinoceros	Y07726.1
	Equus asinus	Donkey	NC_001788.1
	Equus caballus	Horse	NC_001640.1
Primates	Pongo pygmaeus	Bornean Orangutan	D38115.1
	Pongo abelii	Sumatran Orangutan	NC_002083.1
	Hylobates lar	Lar Gibbon	X99256.1
	Gorilla gorilla	Gorilla	D38114.1
	Cercopithecus aethiops	Grivet monkey	AY863426.1
	Macaca sylvanus	Barbary Macaque	NC_002764.1
	H.sapiens	Human	V00662.1
	Papio hamadryas	Baboon	Y18001.1
	Pan troglodytes	Common Chimpanzee	D38113.1
	Pan paniscus	Pigmy Chimpanzee	D38116.1
Artiodactyla	Bubalus bubalis	Buffalo	AY488491.1
	Bos taurus	Cow	V00654.1
	Capra hircus	Goat	AF533441.1
	Ovis aries	Sheep	AF010406.1
	Pantholops hodgsonii	Tibetan Antelope	NC_007441.1
	Capricornis swinhoei	Taiwan serow	NC_010640.1
	Sus scrofa	Wild Boar	AJ002189.1
	Phacochoerus africanus	Common Warthog	NC_008830.1
Erinaceomorpha	Erinaceus europaeus	Hedge Hog	X88898.2
Rodentia	Myoxus glis	Edible dormouse	AJ001562.1
	Sciurus vulgaris	Red Squirrel	AJ238588.1
Carnivore	Canis familiaris	Dog	U96639.2
	Canis lupus chanco	Himalayan Wolf	EU442884.2
	Ailuropoda melanoleuca	Giant Panda	EF212882.1
	Ursus arctos	Brown Bear	AF303110.1
	Ursus maritimus	Polar Bear	AF303111.1
	Ursus thibetanus	Asian Black Bear	DQ402478.1
	Felis catus	Cat	U20753.1
	Panthera pardus	Leopard	EF551002.1
	Panthera tigris	Tiger	EF551003.1
Cetacea	Balaena mysticetus	Bowhead Whale	NC_005268.1
	Eschrichtius robustus	Gray Whale	NC_005270.1
	Eubalaena japonica	North Pacific Right Whale	NC_006931.1
	Platanista minor	Indus river Dolphin	NC_005275.1
	Balaenoptera musculus	Blue Whale	X72204.1
	Balaenoptera physalus	Fin Whale	NC_001321.1
Lagomorpha	Oryctolagus cuniculus	European Rabbit	AJ001588.1

Table S2: Genome sequence details of SARS-CoV-1 and Non-SARS-CoV-1 coronaviruses belonging to Groups I, II and III

**Virus type**	**Genome**	**Abbreviation**	**Accession Number**
SARS CoV-1 Coronaviruses	SARS corona virus SIN2500	SIN2500	AY283794
	SARS corona virus SIN677	SIN2677	AY283795
	SARS corona virus SIN2679	SIN2679	AY283796
	SARS corona virus SIN2748	SIN2748	AY283797
	SARS corona virus SIN2774	SIN2774	AY283798
	SARS corona virus BJ01	BJ01	AY278488
	SARS corona virus BJ02	BJ02	AY278487
	SARS corona virus BJ03	BJ03	AY278490
	SARS corona virus BJ04	BJ04	AY279354
	SARS corona virus HKU-39849	HKU-39849	AY278491
	SARS corona virus TOR2	TOR2	AY274119
	SARS corona virus Urbani	Urbani	AY278741
	SARS corona virus CUHK-W1	CUHKU-W1	AY278554
	SARS corona virus CUHK-Su10	CUHKU-Su10	AY282752
	SARS corona virus GD01	GD01	AY278489
Group I coronavirus	Human corona virus 229E	HCoV-229E	KF514433
	Human corona virus NL63	HCoV-NL63	KF530114
	Transmissible Gastroenteritis virus, Purdue	TGEV	DQ811789
	Transmissible Gastroenteritis virus, strain WH-1	TGEV WH-1	HQ462571
	Porcine epidemic Diarrhea virus	PEDV	NC_003436
	Feline Corona Virus DF-2	FCoV DF-2	DQ286389
Group II coronaviruses	Murine hepatitis virus	JHM MHV-JHM	AC_000192
	Murine hepatitis virus Strain Penn 97-1	MHVP	AF208066
	Murine hepatitis virus Strain ML-10	MHVM	AF208067
	Bovine Corona Virus	BCoV	NC_003045
	Bovine Corona Virus Isolate BCoV-LUN	BCoVL	AF391542
	Porcine hemagglutinating encephalomyelitis,	PHEV	NC_007732
	Porcine hemagglutinating encephalomyelitis, VW572	PHEV -VW572	DQ011855
Group III coronaviruses	Avian infectious bronchitis virus	IBV	NC_001451
	Turkey Corona Virus	TCV	NC_010800

Table S3: Genome sequence details of 30 COVID-19 causing coronavirus sequences and 30 coronavirus sequences not causing COVID-19 (consisting of alpha, beta, gamma and deltacoronaviruses)

**Virus type**	**Genome**	**Abbreviation**	**Accession Number**
SARS-CoV-2 (COVID-19)	hCoV-19/Kuwait/KU09/2020	hCoV19 1	EPI_ISL_416541
	hCoV-19/South Africa/KRISP-011/2020	hCoV19 2	EPI_ISL_421575
	hCoV-19/New Zealand/20CV0181/2020	hCoV19 3	EPI_ISL_548049
	hCoV-19/New Zealand/20CV0636/2020	hCoV19 4	EPI_ISL_707800
	hCoV-19/Uruguay/CEL-M116/2020	hCoV19 5	EPI_ISL_750177
	hCoV-19/Egypt/CCHE57357-P-01/2020	hCoV19 6	EPI_ISL_812850
	hCoV-19/French Guiana/IPP00588/2021	hCoV19 7	EPI_ISL_890352
	hCoV-19/Afghanistan/IMB07966/2020	hCoV19 8	EPI_ISL_1001002
	hCoV-19/Morocco/FMP-82/2021	hCoV19 9	EPI_ISL_1159698
	hCoV-19/Ethiopia/39379/2020	hCoV19 10	EPI_ISL_1170957
	hCoV-19/Hong Kong/HKU-210308-L34/2020	hCoV19 11	EPI_ISL_1197103
	hCoV-19/Guam/GU-CDC-2-3906191/2020	hCoV19 12	EPI_ISL_1273054
	hCoV-19/Bangladesh/BSMMU-11/2020	hCoV19 13	EPI_ISL_1299202
	hCoV-19/Romania/Bucuresti$_4$30063/2021	hCoV19 14	EPI_ISL_1381311
	hCoV-19/Argentina/PAIS-D0091/2021	hCoV19 15	EPI_ISL_1396360
	hCoV-19/Australia/QLD1760/2021	hCoV19 16	EPI_ISL_1396521
	hCoV-19/Papua New Guinea/PNG544/2021	hCoV19 17	EPI_ISL_1424873
	hCoV-19/Uganda/UG337/2020	hCoV19 18	EPI_ISL_1469406
	hCoV-19/Russia/SPE-RII-MH14658S/2021	hCoV19 19	EPI_ISL_1491705
	hCoV-19/Finland/13DS7CG11/2020	hCoV19 20	EPI_ISL_1595812
	hCoV-19/Peru/ARE-UPCH-0391/2021	hCoV19 21	EPI_ISL_1629781
	hCoV-19/Madagascar/IPM-40288/2020	hCoV19 22	EPI_ISL_1660331
	hCoV-19/India/MH-ICMR-NIV-INSACOG-GSEQ-1129/2021	hCoV19 23	EPI_ISL_1704606
	hCoV-19/Chile/AP-78245/2021	hCoV19 24	EPI_ISL_1712369
	hCoV-19/Australia/QLD1850/2021	hCoV19 25	EPI_ISL_1754857
	hCoV-19/Switzerland/SH-ETHZ-530661/2020	hCoV19 26	EPI_ISL_1760974
	hCoV-19/India/CH-ICMR-991/2020	hCoV19 27	EPI_ISL_1761617
	hCoV-19/Italy/ABR-IZSGC-228180/2021	hCoV19 28	EPI_ISL_1785135
	hCoV-19/Germany/SL-SU-10451049/2020	hCoV19 29	EPI_ISL_1786492
	hCoV-19/Brazil/SP-Hemocentro-FMRP-New15/2021	hCoV19 30	EPI_ISL_1786561
Alphacoronavirus	Swine acute diarrhea syndrome coronavirus isolate SADS-CoV/GDWT-P7	Alpha CoV 1	MK994934
	Swine acute diarrhea syndrome coronavirus strain GDLX/2019	Alpha CoV 2	MK651076
	Porcine epidemic diarrhea virus strain COL/Antioquia09831/2015	Alpha CoV 3	MK071636
	Porcine epidemic diarrhea virus strain COL/Antioquia00265/2015	Alpha CoV 4	MK071619
	Swine acute diarrhea syndrome coronavirus isolate 211	Alpha CoV 5	MF769421
	Porcine epidemic diarrhea virus isolate GER/L03210/2019	Alpha CoV 6	LR812931
	Ferret coronavirus isolate FRCoV-NL-2010	Alpha CoV 7	KM347965
Betacoronavirus	Murine hepatitis virus strain A59 isolate WT-MHV P250	Beta CoV 1	MF618252
	BtVs-BetaCoV/SC2013	Beta CoV 2	KJ473821
	Betacoronavirus Erinaceus/VMC/DEU/2012 isolate ErinaceusCoV/2012-174/GER/2012	Beta CoV 3	KC545383
	Murine coronavirus strain JHM.WU	Beta CoV 4	JX169867
	Murine hepatitis virus strain S/3239-17, complete genome.	Beta CoV 5	JQ173883
	Rat coronavirus isolate 681	Beta CoV 6	JF792616
	Murine hepatitis virus strain S	Beta CoV 7	GU593319
	Murine coronavirus RA59/R13	Beta CoV 8	FJ647218
	Murine hepatitis virus strain A59	Beta CoV 9	AY700211
	Murine hepatitis virus strain JHM	Beta CoV 10	AC_000192
Gammacoronavirus	Infectious bronchitis virus strain cK/CH/LDL/091021	Gamma CoV 1	MN509587
	Infectious bronchitis virus isolate ArkGA$_P$60	Gamma CoV 2	MH779859
	Infectious bronchitis virus isolate IBV$_SES_15AB-01$	Gamma CoV 3	MH539771
	Infectious bronchitis virus isolate IBS037A/2014	Gamma CoV 4	MG738154
	Avian coronavirus strain L1148	Gamma CoV 5	KY933090
	Bottlenose dolphin coronavirus HKU22 isolate CF090331	Gamma CoV 6	KF793826
Deltacoronavirus	Porcine coronavirus HKU15 strain OH11846	Delta CoV 1	KT381613
	Porcine deltacoronavirus strain USA/Minnesota/2013	Delta CoV 2	KR265853
	Porcine deltacoronavirus isolate CHN-AH-2004	Delta CoV 3	KP757890
	Porcine coronavirus HKU15 strain PA3148	Delta CoV 4	KJ584358
	Porcine deltacoronavirus 8734/USA-IA/2014	Delta CoV 5	KJ567050
	Porcine coronavirus HKU15 strain HKU15-155	Delta CoV 6	JQ065043
	Bulbul coronavirus HKU11-934	Delta CoV 7	FJ376619

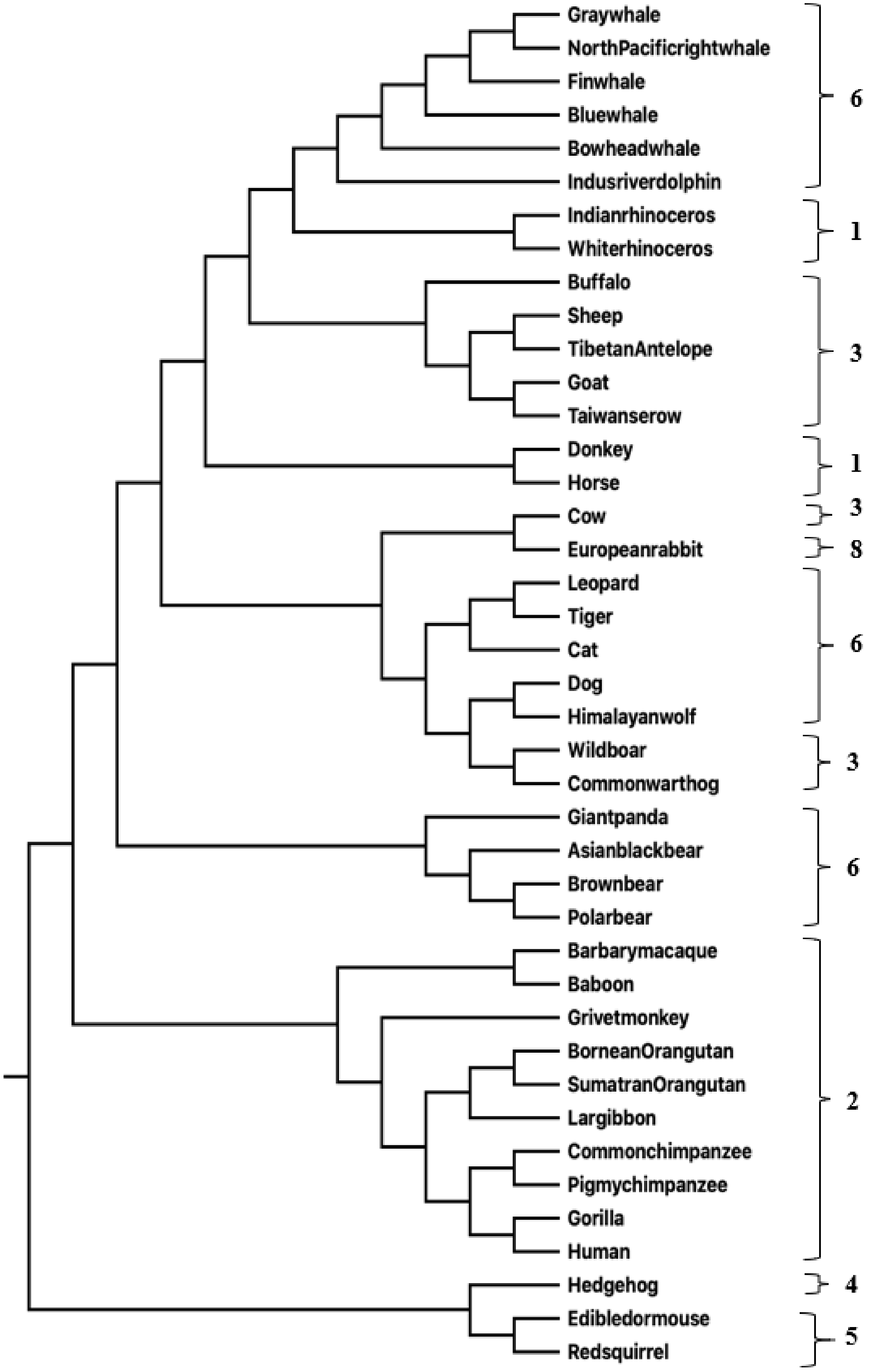

Figure S1: Phylogenetic tree for mammals generated using LZC based distance measure

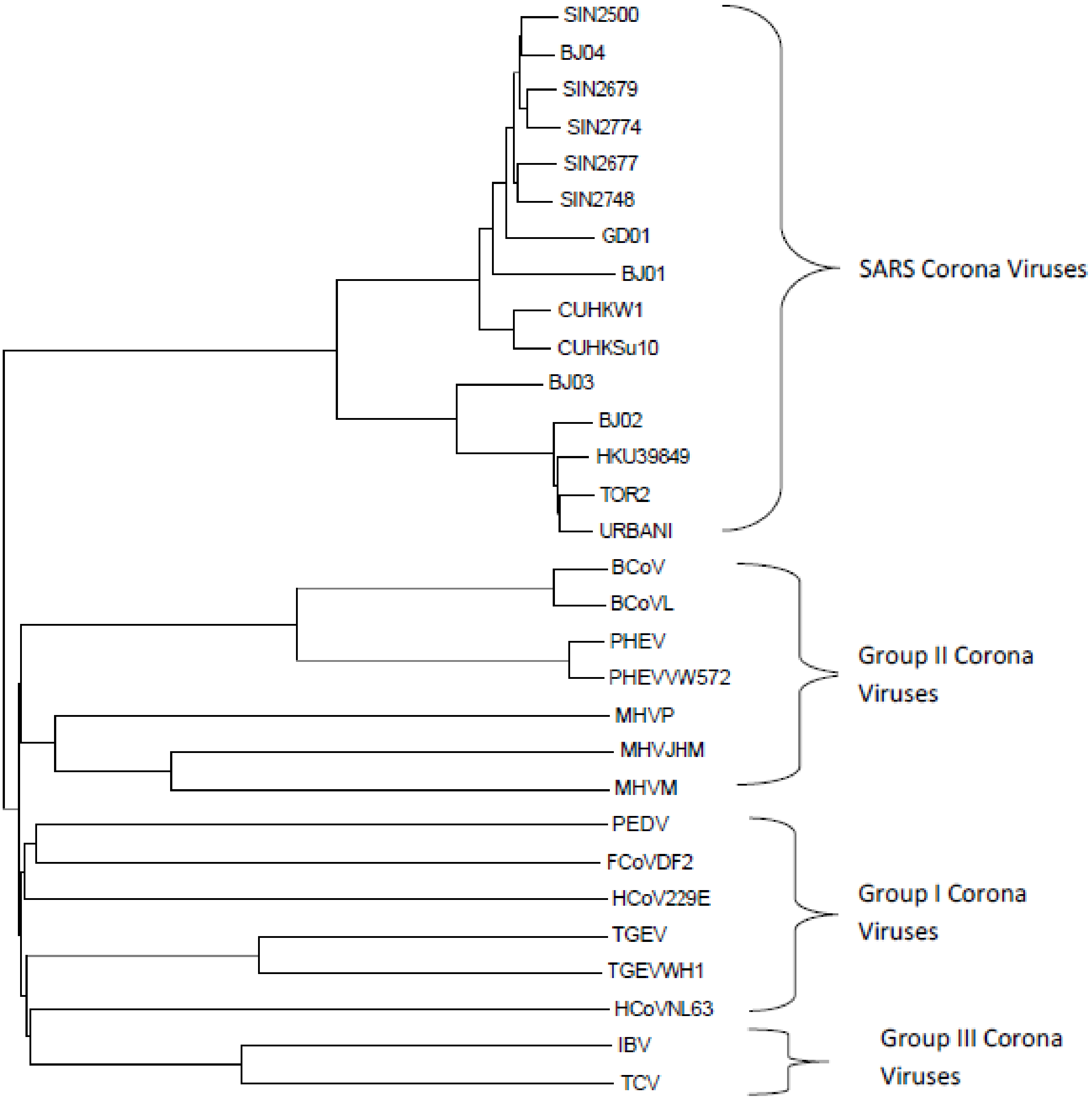

Figure S2: Phylogenetic tree generated for coronaviruses (SARS-CoV-1 and non-SARS-CoV-1) with LZC based distance measure.

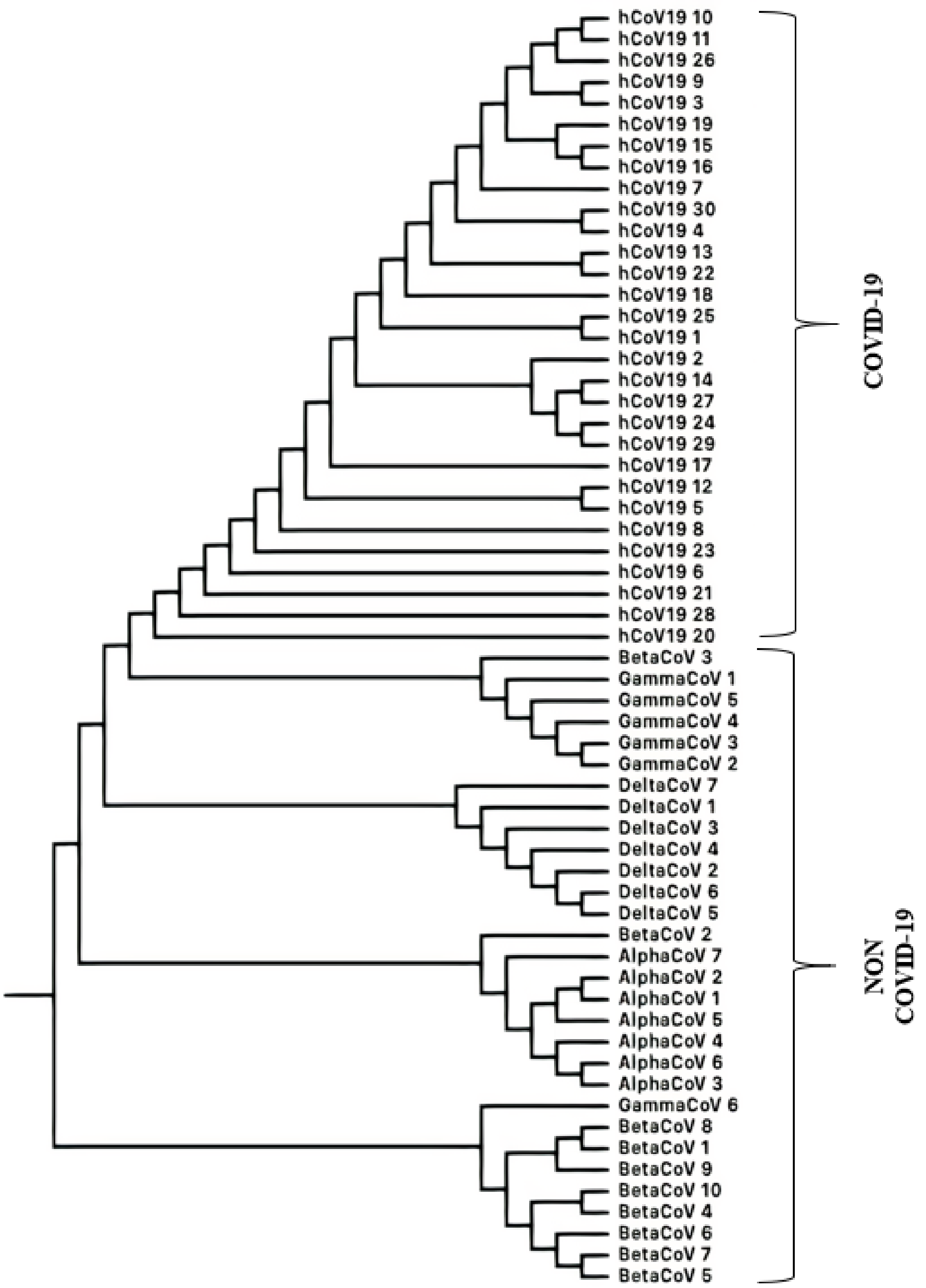

Figure S3: Phylogenetic tree generated for coronaviruses causing COVID-19 and those not causing COVID-19 using LZC based distance measure.

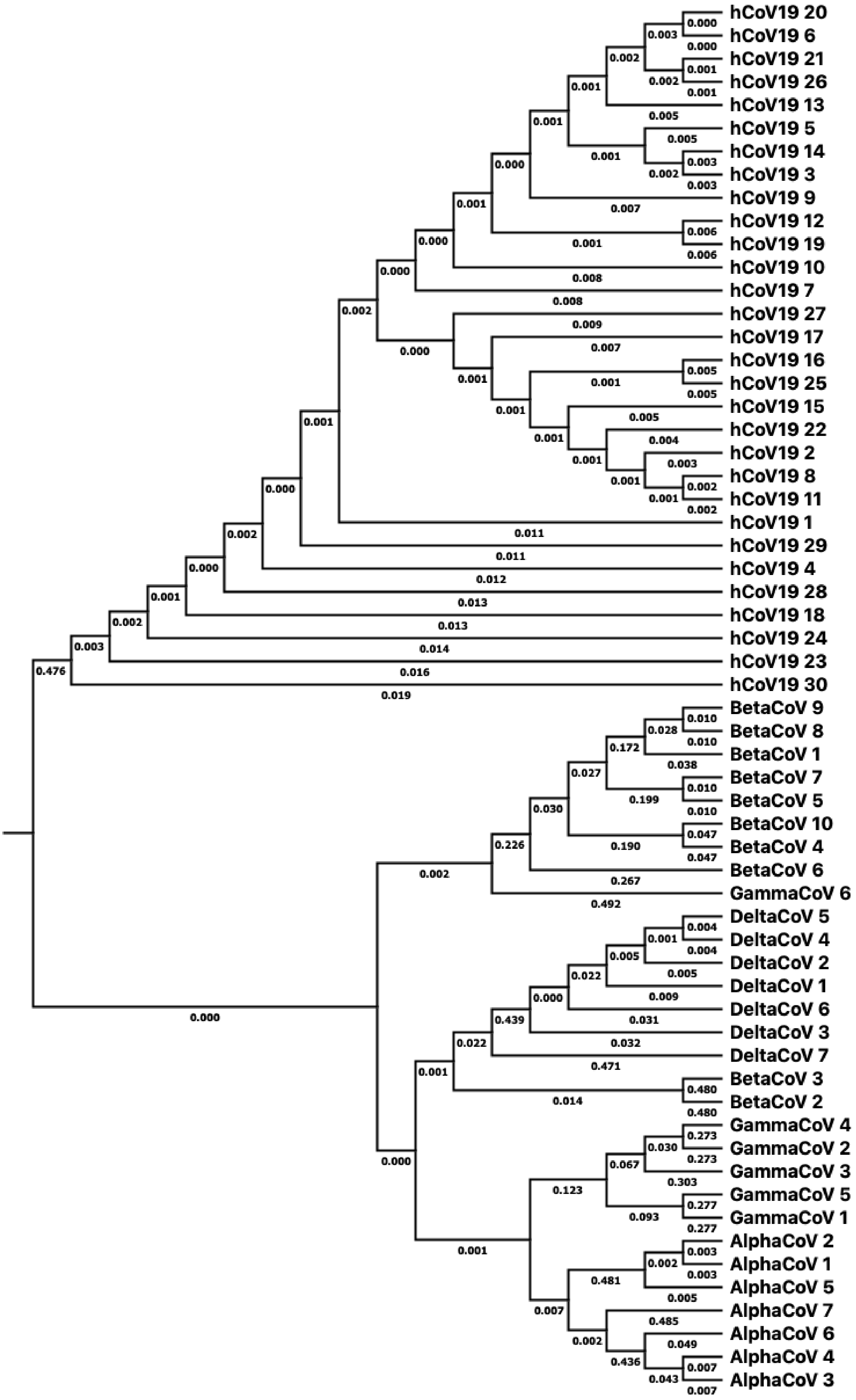

Figure S4: Phylogenetic tree generated for coronaviruses causing COVID-19 and those not causing COVID-19 using ETC based distance measure, with distances displayed.

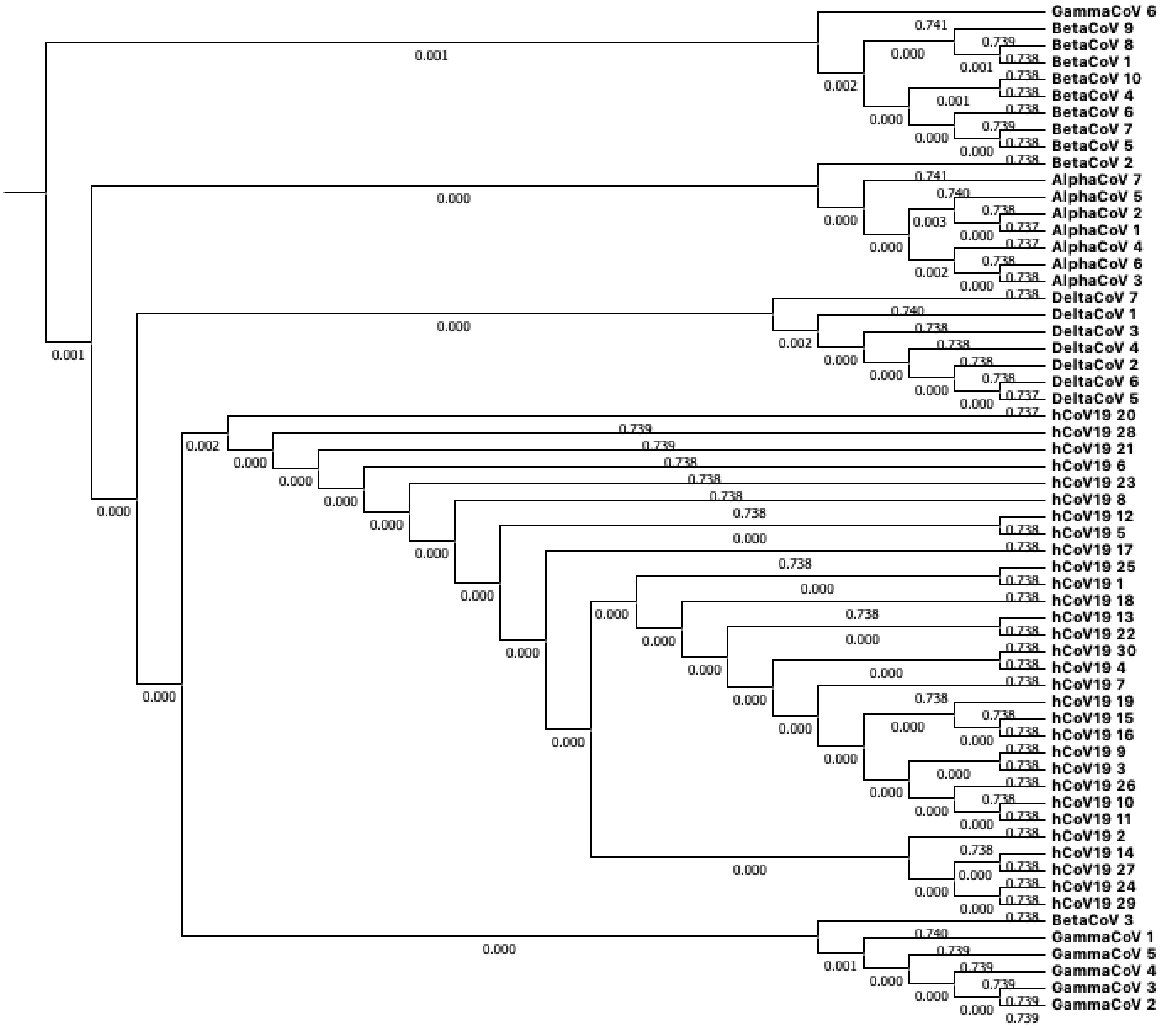

Figure S5: Phylogenetic tree generated for coronaviruses causing COVID-19 and those not causing COVID-19 using LZC based distance measure, with distances displayed.
