# Supplementary material for: Compression-Complexity Measures for Analysis and Classification of Coronaviruses
Source: Entropy (Basel). 2022 Dec 31;25(1):81. doi: 10.3390/e25010081 (PMC9857615; doi:10.3390/e25010081)
Supplement: Supplementary file 1 [file entropy-25-00081-s001.zip › Supplementary_Resource 2.pdf]

Supplementary Information-2 for ‘Compression-complexity measures for analysis and classification of coronaviruses’

December 29, 2022

Compression-Complexity Measures

We provide below a brief description of the two compression-complexity measures that are used in this study:

Lempel-Ziv Complexity or LZC:

Lempel Ziv Complexity or LZC [1] is widely used in a number of applications. To compute LZC of the input sequence $A = \{a_i\}_{i=1}^{i=n} = a_1a_2 \ldots a_n$, it is parsed from left to right and all distinct patterns are counted. This parsing method is proposed in [1] and is also related to the universal source compression algorithm and to the universal compression algorithm in [2].

Following [3], let the input sequence be denoted as $A = a_1a_2 \cdots a_n$. Let $A(p, q)$ denote a substring of A that begins at position p and ends at position q. $V(A)$ denote the set of all substrings $\{A(p, q), p = 1, 2, \cdots n; q \geq p\}$. For example, let $A = abc$, then $V(A) = a, b, c, ab, bc, abc$ (parsing from left to right).

Now, start with $p = 1$ and $q = 1$. A substring $A(p, q)$ is compared with all strings in A $(A(p, q-1))$ (Let $V(A(1, 0)) = \{\}$, the empty set). If $A(p, q)$ is present in $V(A(1, q-1))$, then increase q by 1 and repeat the process. If the substring is not present, then place a dot after $A(p, q)$ to indicate the end of a new component, set $p = q + 1$, increase q by 1, and the process continues. This parsing procedure continues until $q = n$. For example, the sequence ‘*ccagcagc*’ is parsed as ‘*c.ca.g.cagc.*’. By convention, a dot is placed after the last element. LZC of A is defined as the number of dots as this is the number of distinct words. Here, $LZ(A) = 4$. To allow us to compare the LZ complexity of sequences of different lengths, a normalized measure is proposed [4]:

$$C_{LZ} = (c(n)/n)log_{\alpha}n.$$

where α denotes the number of unique symbols in the input sequence A.

Effort-To-Compress Complexity or ETC

Effort-To-Compress complexity or ETC, proposed in [5], measures the effort needed by a lossless compression algorithm to compress the input sequence. Non-sequential Recursive Pair Substitution (NSRPS) [6] is the lossless compression algorithm that is employed by ETC. To compute ETC, the input sequence is parsed from left to right and that pair of symbols that has the maximum frequency is determined. This pair is replaced by a new distinct symbol throughout the sequence. In the next iteration, the same process is repeated until the sequence length reduced to 1 or a constant sequence. As an example, let the input sequence be $A = 11001010$. The pair ‘10’ is the most frequently occurring pair (when compared with the pairs ‘00’, ‘01’ and ‘11’). It is replaced with ‘2’ to yield the new sequence 12022. In the next iteration, 12022 is transformed to 3022. This process is continued. Thus, for the above example, the algorithm transforms the input sequence $11001010 \mapsto 12022 \mapsto 3022 \mapsto 422 \mapsto 52 \mapsto 6$, and thus takes 5 iterations to halt. ETC is defined as the number of iterations needed to convert the input sequence to constant sequence and in this example, the value is 1. In order to be able to compare ETC across sequences of different lengths, it is normalized by dividing by the value $L-1$ where L is the length of the input sequence (since the maximum value of ETC for a sequence of length L is $L-1$). Thus, the normalized value of ETC always lies between 0 and 1. For this example, the normalized ETC value is $\frac{5}{8-1} = 5/7 = 0.7143$.

We provide open implementation of ETC (in MATLAB® and Python) for free download and use (for research and academic purposes only). Please visit:
https://sites.google.com/site/nithinnagaraj2/journal/etc.

S1 SVM, KNN and Linear Discriminant

S1.1 Support Vector Machines (SVM)

Support Vector Machine (SVM) is a popular Supervised Machine Learning algorithm that is mainly used for classification problems but can also be used for regression problems. The SVM algorithm aims to find the best line or decision boundary (with maximum margin [7]) for categorizing n-dimensional space so that we can effectively place new data points in the right category in the future. This best decision boundary is called a "hyperplane". SVM selects the extreme points/vectors that aid in the development of the hyperplane. These extreme cases are referred to as support vectors, and the algorithm is known as the Support Vector Machine. The dimensions of the hyperplane are determined by the number of features present; for two features, the hyperplane is a straight line; for three features, it is a two-dimensional plane. We always obtain a hyperplane with the greatest possible margin using SVM.

Types of SVM:

- Linear SVM: Linear SVM is used for linearly separable data, and classifier is used called as Linear SVM classifier.
- Non-linear/quadratic SVM: Non-Linear SVM is used for non-linearly separated data, which means if a dataset cannot be classified by using a hyperplane, the classifier used is called as Non-linear SVM classifier. To this end, kernels are utilized.

S1.2 K-Nearest Neighbors (KNN)

K-Nearest Neighbors (KNN) is a simple Machine Learning algorithm that uses the Supervised Learning technique. The KNN algorithm stores all available data and classifies new data points based on similarities. This means that when new data is collected, it can be easily categorized into a well-suited category using the KNN algorithm. The KNN algorithm can be used for both regression and classification, but it is most often used for classification problems. KNN is a non-parametric algorithm, which means it makes no assumptions about the underlying data. KNN works as follows: first, we choose the number K (an odd number) of neighbors, then determine the K nearest neighbors (of the test data point) based on the Euclidean distance, and count the number of data points in each group of these K neighbors. Finally, assign the label for the test data point to that class label which has maximum representation in these K nearest neighbours (majority voting scheme).

S1.3 Linear Discriminant Analysis (LDA)

Linear Discriminant Analysis is a statistical method that uses dimensionality reduction techniques to map higher dimensional data onto a lower dimensional space to aid supervised classification problems [8]. Dimensionality reduction helps in discovering parameters relating objects and their corresponding groups, thus creating efficient classification models for multiclass problems. The main idea behind LDA is to find a projection to a line that separates different classes. It aims to maximize the distance between the means of the classes and to minimize the variance within individual classes, thus increasing the separation between the classes leading to better classification.

References

[1] A. Lempel and J. Ziv, "On the complexity of finite sequences," *IEEE Transactions on information theory*, vol. 22, no. 1, pp. 75–81, 1976.

[2] J. Ziv and A. Lempel, "A universal algorithm for sequential data compression," *IEEE Transactions on information theory*, vol. 23, no. 3, pp. 337–343, 1977.

[3] J. Hu, J. Gao, and J. C. Principe, "Analysis of biomedical signals by the lempel-ziv complexity: the effect of finite data size," *IEEE Transactions on Biomedical Engineering*, vol. 53, no. 12, pp. 2606–2609, 2006.

[4] M. Aboy, R. Hornero, D. Abásolo, and D. Álvarez, "Interpretation of the lempel-ziv complexity measure in the context of biomedical signal analysis," *IEEE Transactions on Biomedical Engineering*, vol. 53, no. 11, pp. 2282–2288, 2006.

[5] N. Nagaraj, K. Balasubramanian, and S. Dey, "A new complexity measure for time series analysis and classification," *The European Physical Journal Special Topics*, vol. 222, no. 3-4, pp. 847–860, 2013.

[6] W. Ebeling and M. A. Jiménez-Montaño, "On grammars, complexity, and information measures of biological macromolecules," *Mathematical Biosciences*, vol. 52, no. 1, pp. 53–71, 1980.

[7] B. E. Boser, I. M. Guyon, and V. N. Vapnik, "A training algorithm for optimal margin classifiers," in *Proceedings of the fifth annual workshop on Computational learning theory*, pp. 144–152, 1992.

[8] G. James, D. Witten, T. Hastie, and R. Tibshirani, *An Introduction to Statistical Learning*, vol. 112. Springer, 2013.
